# Applications of Robotic Exoskeletons as Motion-Assistive Systems in Cancer Rehabilitation

**DOI:** 10.34133/research.0855

**Published:** 2025-11-21

**Authors:** Yisheng Chen, Guanghui Wu, Qiangqiang Wang, Ye Ding, Li Wu, Haojun Shi, Zijin Sun, Zemin Ou, Yunxuan Miao, Xiuhui Ji, Ke Wu, Zhiwen Luo, Weijian Chen, Zhijie Zhao, Chen Hua, XianGuo Fu, Yazhen Zhang, Ruogu Chen, Shiwei He, Lizhi Li, Shiyi Chen, Lei Huang, Lihua Dai

**Affiliations:** ^1^ Ningde Municipal Hospital of Ningde Normal University, Ningde, Fujian Province, China.; ^2^ Department of Neurosurgery, Ningde Clinical Medical College, Fujian Medical University, Ningde, Fujian Province, China.; ^3^ Fujian Key Laboratory of Toxicant and Drug Toxicology, Medical College, Ningde Normal University, Ningde, Fujian, China.; ^4^ Tongji Medical College, Huazhong University of Science and Technology, Wuhan, China.; ^5^ Yrobot Inc., Kunming, Yunnan, China.; ^6^ State Key Laboratory of Primate Biomedical Research; Institute of Primate Translational Medicine, Kunming University of Science and Technology, Kunming, Yunnan 650500, China.; ^7^ Yunnan Key Laboratory of Primate Biomedical Research, Kunming, Yunnan 650500, China.; ^8^ Faculty of Chinese Medicine and State Key Laboratory of Quality Research in Chinese Medicines, Macau University of Science and Technology, Macau, MacauSAR, China.; ^9^ Beijing University of Chinese Medicine, Beijing, China, Luoyang, Henang, China.; ^10^ Institute of Chinese Materia Medica, China Academy of Chinese Medical Sciences, Beijing 100700, China.; ^11^ Henan University of Science and Technology, Luoyang, Henang, China.; ^12^ Department of Pediatrics, School of Pediatrics, Nanjing Medical University, Nanjing, China.; ^13^ Kangwon National University, Gangwon-do, Korea.; ^14^ Department of Sports Medicine, Huashan Hospital, Fudan University, Shanghai 200040, China.; ^15^ Guangzhou University of Chinese Medicine, Guangzhou 510405, Guangdong, China.; ^16^ Department of Plastic and Reconstructive Surgery, Shanghai Ninth People’s Hospital, Shanghai JiaoTong University School of Medicine, Shanghai, China.; ^17^ Faculty of Science, Universiti Malaya, Kuala Lumpur, Malaysia.; ^18^ School of Physical Education, Ningde Normal University, Ningde, Fujian 352300, China.; ^19^ Animal Medicine, College of Life Sciences, Longyan University, Longyan, China.; ^20^ Institute of Population Medicine, School of Public Health, Fujian Medical University, Fuzhou 350108, Fujian, China.; ^21^ Department of Pediatric Surgery, Shengli Clinical Medical College of Fujian Medical University, Fuzhou, Fujian 350001, China.; ^22^ Department of Pediatric Surgery, Affiliated Provincial Hospital of Fuzhou University, Fuzhou, Fujian 350001, China.; ^23^ Department of Molecular, Cell, and Cancer Biology, University of Massachusetts Chan Medical School, Worcester, MA 01605, USA.; ^24^ Emergency and Intensive Care Unit, Shidong Hospital affiliated to University of Shanghai for Science and Technology, Shanghai, China.

## Abstract

Robotic exoskeletons have emerged as promising motion-assistive technologies to meet the growing demand for structured, personalized cancer rehabilitation. This review critically examines their application in enhancing functional recovery, alleviating cancer-related fatigue, and improving quality of life among cancer patients and survivors. We first outline current evidence-based exercise strategies in cancer rehabilitation, followed by an analysis of how robotic exoskeletons complement and extend these approaches by restoring mobility, strengthening musculature, and supporting psychological well-being. Additionally, we assess how well they combine with multimodal interventions like nutritional, psychological, and digital therapeutics and their benefits. The convergence of artificial intelligence, wearable sensors, and telemedicine is also reshaping the landscape of remote, adaptive rehabilitation. Despite encouraging preliminary data, widespread clinical adoption remains limited due to challenges related to cost, exoskeleton system accessibility, safety, and long-term efficacy. We call for large-scale clinical trials, interdisciplinary collaboration, and policy reforms to promote equitable access to robotic-assisted rehabilitation. Collectively, this review offers a comprehensive perspective on the technical principles, therapeutic potential, and future directions of robotic exoskeletons as innovative tools in cancer recovery.

## Introduction

Cancer remains a major global health burden, with 19.3 million new cases and 9.9 million deaths reported in 2020 [1]. In most developed countries, the 5-year survival rate for common cancers such as breast and prostate cancer now exceeds 70%, although the aggregate 5-year survival across all major cancer types remains lower [2]. Current treatment protocols yield better outcomes as compared to the past. However, many patients continue to experience treatment-related complications like pain, fatigue, anxiety, depression, and sleep disturbances. These commonly afflict a patient’s cognitive, psychosocial, and physical functioning. Notably, these complications decrease health-related quality of life (HRQoL). Furthermore, oncologic therapies contribute to long-term cardiovascular risk, prompting a shift toward comprehensive supportive care and validated rehabilitation strategies [3]. One Health is especially vulnerable in cancer survivors, particularly those undergoing chemotherapy, radiotherapy, or hormonal therapies such as aromatase inhibitors and gonadotropin-releasing hormone agonists. These treatments can compromise bone integrity through direct cytotoxic effects, hormonal suppression, or vascular damage. Prolonged glucocorticoid use, androgen deficiency, and ovarian dysfunction further exacerbate the risk of osteoporosis and fractures. In parallel, cancer-related malnutrition often results in decreased intake of calcium and vitamin D, thereby worsening skeletal fragility [4]. Therefore, a personalized assessment of bone health and fracture risk is critical prior to initiating cancer treatment, particularly in patients at risk of premature menopause or extended steroid therapy.

This study focuses on the role of robotic exoskeletons in cancer rehabilitation, particularly evaluating their ability to restore physical function, reduce cancer-related fatigue (CRF), and improve quality of life. CRF is an extreme fatigue symptom that cannot be relieved by regular rest, damaging bodily functions and reducing quality of life. Research has shown that CRF is closely related to inflammatory response, energy metabolism disorders, and muscle function degradation. Resistance training (RT) can alleviate CRF, especially when the training cycle exceeds 6 weeks. The combination of robot exoskeleton and RT has advantages in reducing fatigue, enhancing muscle strength, and improving quality of life. Cancer survivors often develop sarcopenia due to treatment, which affects their ability to perform daily activities. The robot exoskeleton provides a safe and effective exercise program through dynamic weight-bearing support and gait training, reducing the risk of injury and supporting muscle function recovery [5]. Thus, the research objectives of robotic exoskeletons in cancer rehabilitation include evaluating their actual effects on physical function restoration, exploring their role in alleviating CRF, and verifying their comprehensive benefits in improving patients’ quality of life [6]. In the future, by combining genomic data and clinical features of cancer patients, robotic exoskeletons can be customized to meet the individual needs of patients, providing personalized rehabilitation solutions. This method not only improves the effectiveness of rehabilitation but also conforms to the development trend of precision medicine in oncology.

### Robotic exoskeletons in cancer rehabilitation

**Fig. 1.**
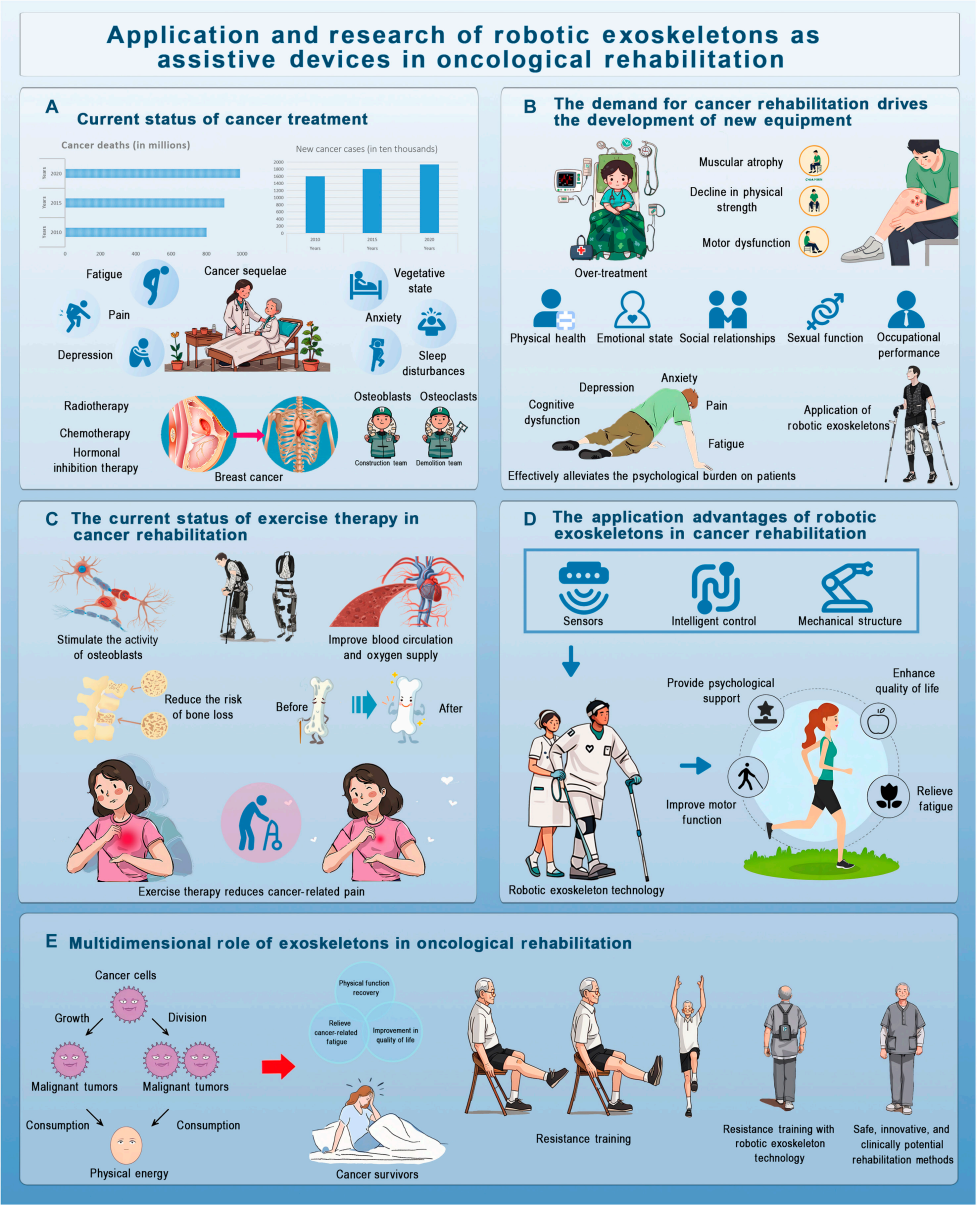
Overview of robotic exoskeletons in cancer rehabilitation. (A) Current cancer treatment status. This section shows cancer statistics, including mortality and incidence rates, and highlights common side effects like fatigue, pain, depression, and osteoblast dysfunction, particularly in breast cancer patients. (B) Demand for cancer rehabilitation drives equipment development. The increasing need for cancer rehabilitation due to muscular atrophy, physical decline, cognitive dysfunction, and pain is driving the development of robotic exoskeletons aimed at improving both physical and emotional well-being. (C) Exercise therapy in cancer rehabilitation. Exercise therapy’s benefits, such as stimulating osteoblast activity, improving circulation, and reducing bone loss, are shown, with a comparison of CRP levels before and after exercise to demonstrate pain reduction. (D) Advantages of robotic exoskeletons in cancer rehabilitation. This section emphasizes the technological benefits of robotic exoskeletons, including sensors, intelligent control, and mechanical structures, which aid in motor function recovery, alleviate fatigue, and provide psychological support. (E) Multidimensional role of exoskeletons. Exoskeletons’ broader role in improving physical function and enhancing the quality of life for cancer survivors is explored, highlighting their integration into resistance training (RT) programs for safe, innovative rehabilitation.

Rehabilitation, as defined by the World Health Organization, aims to optimize functional capacity and reduce disability. In cancer care, comprehensive rehabilitation, including physical therapies, pharmacological support, psychological interventions, and social reintegration strategies, is now essential and integrated into clinical guidelines. However, many cancer patients continue to face functional impairments such as sarcopenia and mobility issues after treatment, necessitating individualized, multidisciplinary rehabilitation across different care settings (Fig. 1 and Table 1) [6,7]. Cancer survivors often experience diminished HRQoL due to pain, fatigue, and psychosocial distress. Structured interventions like physical activity and dietary optimization improve functional outcomes and reduce recurrence risk [8], while ongoing management of chronic conditions such as cardiovascular disease and metabolic syndrome is crucial. Mental health symptoms can persist long-term, and psychosocial therapies are effective in addressing them [9]. To meet these complex needs, technological innovations are increasingly integrated into cancer rehabilitation. Among them, robotic exoskeletons offer a promising solution for motor impairments (Table 1). By offering real-time assistance, robotic exoskeletons support motor recovery and encourage autonomous movement, shifting cancer rehabilitation toward precision, individualized care [10].

**Table 1.** Comparison of existing cancer rehabilitation methods

Rehabilitation method	Implementation mode	Applicable stage	Advantages	Limitations
Traditional physical therapy	Manual rehabilitation, equipment-assisted	Post-surgery recovery, long-term survival	Low cost, mature technology	Dependent on therapists, lack of personalization
Robot-assisted training	Exoskeleton systems, intelligent rehabilitation robots	Post-surgery recovery	Precise control, quantifiable assessment	High equipment cost, high technical requirements
Digital rehabilitation technology	Remote guidance, wearable exoskeleton system monitoring	Long-term survival	Convenient, can be implemented remotely	Relies on patient initiative, limited data accuracy
Multimodal intervention	Exercise + Psychological support + Nutritional intervention	All stages	Comprehensive improvement of physical and psychological state	Complex to implement, high resource demand

### Current application of exercise therapy in cancer rehabilitation

Cancer rehabilitation adopts a holistic, patient-centered approach that addresses physical, psychological, and functional impairments. In recent years, the field has expanded rapidly, with over 360 studies emphasizing the shift toward individualized, multidimensional care aimed at restoring health and improving long-term quality of life [11]. Exercise therapy is central to this paradigm. Mechanistically, it promotes bone health by stimulating osteoblast activity through mechanical loading and enhancing bone metabolism via improved circulation and oxygenation. Resistance and high-impact exercises are especially effective in promoting bone formation and mitigating bone loss by down-regulating pro-resorptive mediators such as RANKL (Fig. 1).

Cancer-related pain (CRP) remains a common and debilitating symptom among patients and survivors. Although opioids and adjunctive medications remain first-line treatments, concerns about side effects and long-term safety have driven interest in nonpharmacological alternatives. Within multimodal pain management frameworks, exercise has emerged as a promising intervention. It has multi-system effects that are biological, psychological and functional and carries little risk.

Preliminary studies support the use of exercise to alleviate CRP, although current evidence is heterogeneous and of moderate quality. Nonetheless, its safety, accessibility, and integration potential within interdisciplinary care make it a valuable adjunct. Future research should refine exercise prescriptions and explore its synergy with other nonpharmacological approaches, advancing standardized, personalized pain management in oncology care.

### Advantages of robotic exoskeletons in cancer rehabilitation

Robotic exoskeletons have shown increasing promise in cancer rehabilitation by improving motor function, mitigating treatment-related side effects, and enhancing psychological well-being, particularly in patients with muscle atrophy and mobility impairments [12].

According to clinical studies, the upper-limb exoskeleton training improves strength and joint mobility as measured by Timed Up and Go (TUG) test in post-surgical breast cancer survivors. Similarly, use after radiotherapy reduces muscle atrophy, prevents osteoporosis, and enhances joint function. Despite these benefits, barriers such as high cost, limited accessibility, and patient adaptability remain. Future research should prioritize cost reduction, user-friendly design, and broader clinical validation.

Exoskeletons also help manage side effects from chemotherapy and radiotherapy, including chemotherapy-induced peripheral neuropathy (CIPN), fatigue, and reduced exercise tolerance. By improving gait, posture, and circulation, these devices alleviate numbness and pain, support neuromuscular recovery, and reduce fall risk [13]. The combination of passive and active movement training prevents functional decline due to immobility and supports safer rehabilitation environments.

Psychologically, robotic assistance contributes to emotional resilience. Enhanced physical function and real-time feedback improve motivation, treatment adherence, and confidence in recovery. Exoskeletons can help reduce the effect of immunotherapy-related toxicities like fatigue and weakness due to cytokine release syndrome by supporting movement and preventing joint stiffness.

To sum up, robotic exoskeletons provide various benefits which include physical, functional, and psychological benefits; thereby making it a useful invention for cancer rehabilitation. Continuous effort is required to enhance safety, accessibility, and clinical integration of all patient demographics.

## Advances and Challenges in Cancer Rehabilitation

### Overview of evidence-based cancer rehabilitation

**Fig. 2.**
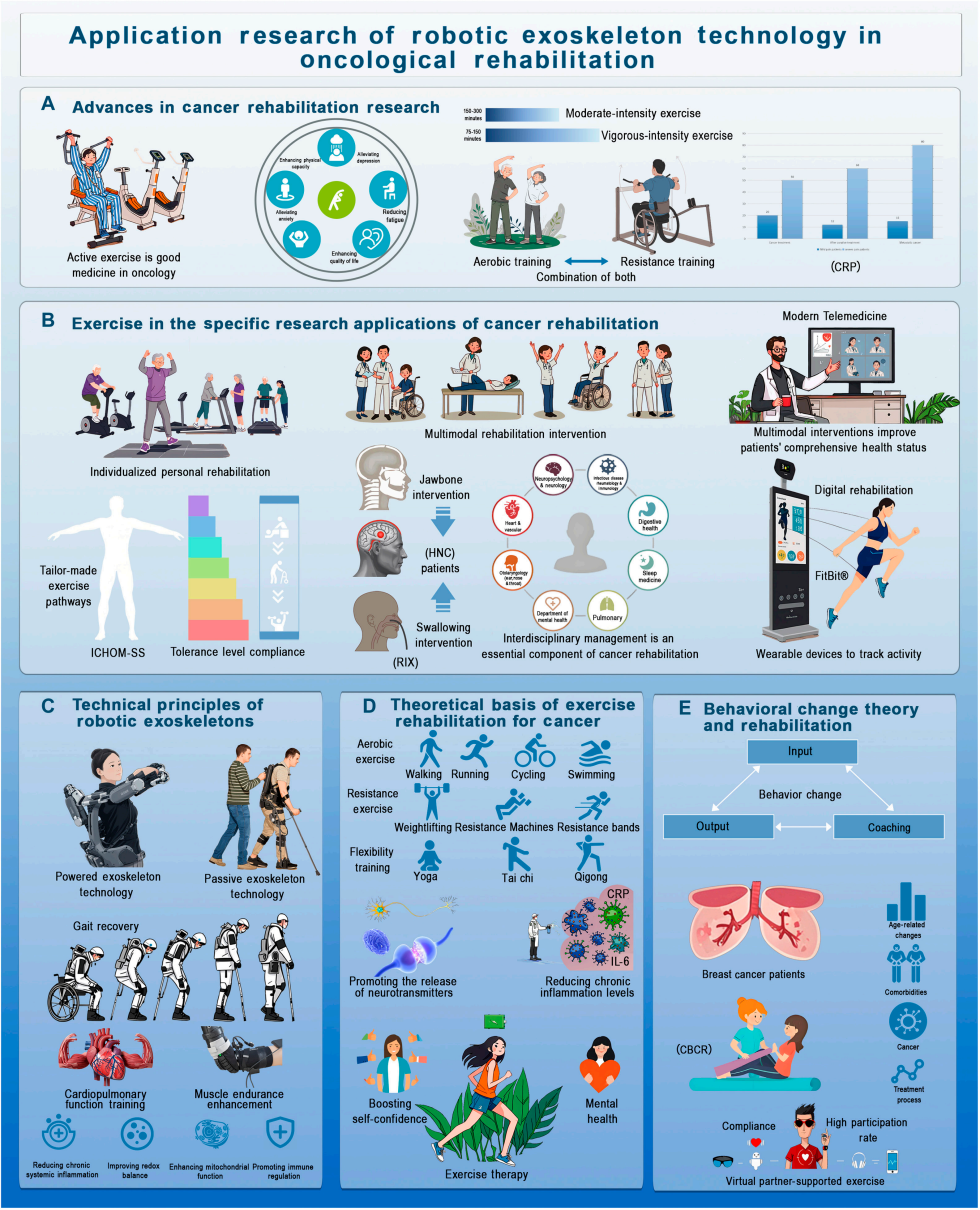
Robotic exoskeleton technology in cancer rehabilitation. (A) Advances in cancer rehabilitation research. This section highlights the benefits of active exercise in cancer rehabilitation, comparing moderate- and vigorous-intensity exercises. It discusses integrating aerobic and resistance training to combat cancer-related fatigue (CRF) and improve physical activity outcomes. (B) Exercise in cancer rehabilitation applications. Various rehabilitation approaches are explored, such as personalized exercise plans and multimodal interventions like jawbone treatments for head and neck cancer (HNC) and swallowing therapies (RIX). The section also covers the integration of telemedicine, digital rehab, and wearable exoskeleton systems (e.g., Fitbit) to track activity and monitor patient health, improving overall outcomes. (C) Technical principles of robotic exoskeletons. This section details the technology behind robotic exoskeletons, distinguishing between powered and passive types. It focuses on their role in gait recovery and muscle endurance, supporting cardiovascular and musculoskeletal rehabilitation. (D) Theoretical basis of exercise rehabilitation for cancer. The types of exercises foundational to cancer rehabilitation are discussed, including aerobic activities, RT, and flexibility exercises like yoga and tai chi. The section also links exercise therapy to physiological benefits such as improved neurotransmitter release, reduced inflammation, and better mental health and self-confidence. (E) Behavioral change theory in rehabilitation. This section examines the role of behavioral change theories in enhancing rehabilitation. It describes how coaching and input–output processes influence patient compliance with exercise protocols, highlighting the importance of high participation rates and virtual partner-supported exercises, particularly for breast cancer patients. These strategies contribute to improved treatment outcomes.

Exercise-based rehabilitation has become a cornerstone of supportive care for cancer patients and survivors, with growing evidence supporting its benefits for physical function, psychosocial well-being, fatigue reduction, and cardiovascular health (Fig. 2) [14]. Meta-analyses confirm that regular physical activity improves quality of life, alleviates depression and anxiety, and mitigates treatment-related side effects. Evidence also supports improvements in sleep quality and bone health [15]. Despite these benefits, implementation gaps remain. Barriers include limited referral rates, inadequate infrastructure, and geographic constraints, which contribute to poor accessibility. In addition, it has been shown that the adherence of patients is sub-optimal since about 42% of people stop following the home-based program. Further, conventional rehabilitation approaches may not provide sufficient motor assistance to patients suffering from severe sarcopenia or post-treatment disability. These limitations underscore the need for adaptive, assistive technologies such as robotic exoskeletons.

To address variability in patient needs, the American College of Sports Medicine (ACSM) issued tailored guidelines for cancer rehabilitation in 2019, recommending 150 to 300 min of moderate-intensity or 75 to 150 min of vigorous exercise per week, alongside twice-weekly RT [16]. However, these regimens may be challenging for patients with CRP, fatigue, or neurological complications. CRP affects nearly 38% of cancer patients and survivors, with prevalence rising to 66% in advanced disease. It may result from tumor infiltration, nerve injury, or treatment-related damage. The PERCS triage system enhances exercise adherence through personalized pathways, increasing participation in aerobic and resistance exercises and improving physical function, supporting patient-centered cancer rehabilitation. While opioids and other pharmacological agents remain standard of care, they often provide incomplete relief and carry risks of dependency and adverse effects. As a result, exercise-assisted approaches are increasingly recommended to reduce opioid use and improve functional outcomes through multimodal and integrative approaches.

#### Personalized exercise-based strategies

Physical exercise improves cancer outcomes including recurrence, survival, pain, and daily function (Fig. 2). A Cochrane review found that exercise programs incorporating goal setting, graded activities, and behavioral guidance help patients maintain recommended activity levels [17]. Behavioral graded activity (BGA), based on operant conditioning, uses personalized plans to gradually increase meaningful activities, enhancing adherence and rehabilitation. Various interventions like exercise, cognitive therapy, and hydrotherapy improve HRQoL, which encompasses physical and mental health perceptions. For example, progressive shoulder strengthening improved pain and function in spinal accessory nerve paralysis patients [18]. Common tools like the TUG and handgrip strength (HGS) tests assess physical function but do not fully capture HRQoL improvements; however, indicators such as gait speed and HGS predict survival and are key rehabilitation markers [19]. Licht et al. showed HRQoL improvements using EORTC QLQ-C30, suggesting that rehabilitation should consider individual differences. Rathore et al. highlighted the value of Patient-Reported Outcome Measures for tailoring interventions, with early comprehensive approaches yielding better outcomes. EORTC QLQ-C30 is widely used across cancer types in trials, with normative data supporting comparisons; the EORTC Survivorship Questionnaire offers more specific HRQoL assessments but needs refinement. The International Consortium for Health Outcomes Measurement (ICHOM) developed oncology standard sets (ICHOM-SS) focusing on key domains and risk adjustments [20]. Exercise effects on cardiovascular fitness vary by chemotherapy type and timing: Anthracycline chemotherapy reduces fitness during treatment but improves post-chemotherapy; noncardiotoxic therapies show less timing effect, indicating that exercise should be tailored accordingly.

#### Multimodal integrated rehabilitation approaches

Recent studies examined integrating personalized intervention plans (PIPs) with BGA for cancer patients’ post-treatment sequelae, mental health, quality of life, and physical activity. Meta-analysis of 33 studies with 14 outcomes showed that PIP + BGA reduced fatigue, anxiety, depression, psychological distress, and social dysfunction while improving physical activity versus waitlist control [21].

Multimodal rehabilitation includes therapeutic exercise, strength training, muscle stimulation, cognitive behavioral therapy (CBT), biofeedback, and yoga (Fig. 2). For head and neck cancer (HNC), combining swallowing and mandibular interventions yields better short-term outcomes than single therapies; referral to speech therapy and acupuncture can reduce radiotherapy-induced inflammation [22]. Although evidence during radiation therapy is limited, combining swimming with RT aids recovery. Other cancers benefit from exercise, nutrition, and psychological interventions to enhance recovery and reduce care burden. A pilot study suggests that acupuncture may alleviate chemotherapy-induced neuropathy pain, although larger studies are needed to confirm long-term benefits.

In postoperative settings, interventions include oral and mandibular exercises, swallowing training, Qigong, and transcutaneous electrical nerve stimulation (TENS) [23]. Multidisciplinary rehabilitation with physical and occupational therapists is critical. Supportive psychotherapy and family education improve emotional stabilization; combined psychotherapy and speech therapy after laryngectomy enhance acceptance and well-being [24]. Cardiac oncology rehabilitation (CORE), integrating exercise, nutrition, and psychological support, outperforms exercise alone, especially in metabolic or cardiovascular toxicity cases, although implementation is limited; remote delivery could broaden access [25]. Forest bathing improved quality of life, fatigue, and anxiety in gynecological cancer rehabilitation, showing promise as complementary therapy.

Community rehab for breast cancer shows that occupational rehab helps under 60 return to work, highlighting the need for personalized integrated approaches [26]. Physical and occupational therapy improve HRQoL in elderly survivors addressing physical and social challenges [27]. The CaRE@ELLICSR project combining education and physical training demonstrated sustained behavioral engagement 3 months post-intervention [28]. Additionally, adequate intake of calcium, vitamin D, and protein remains essential to support musculoskeletal resilience during recovery [29]. Telemedicine-guided rehabilitation, when maintained for 12 weeks or longer, has shown feasibility for remote cardiovascular care [30].

Exercise rehabilitation offers short-term functional gains in survivors, but sustaining benefits is challenging due to motivation, ongoing treatments, and disease progression. Nonetheless, exercise can improve survival and reduce recurrence. The International Bone Metastasis Exercise Working Group recommends personalized programs including aerobic, resistance, flexibility, and balance training under healthcare supervision to improve function and reduce complications [31]. Remote and hybrid models expand access, allowing personalized pacing and lowering care barriers.

#### Emerging technologies in remote cancer rehabilitation

Telemedicine has revolutionized remote cancer rehabilitation by incorporating cognitive therapy, virtual exercise programs, and vocational counseling, making rehabilitation accessible to a wider patient population [32]. Home-based rehabilitation programs, supported by assistive technologies such as robotic exoskeletons, offer a flexible and safe alternative, especially for patients unable to access in-person care. While home settings lack full outpatient facilities, they allow for customizable frequency and intensity of interventions, making rehabilitation more adaptable to individual needs. Additionally, remote platforms utilizing mobile apps, video consultations, and wearable sensors enable real-time monitoring and intervention adjustments, substantially improving patient access and engagement, especially in underserved areas. Remote symptom monitoring, combined with cognitive rehabilitation and psychological support, further enhances patients’ quality of life, especially in managing fatigue, pain, and cognitive impairments [33].

Emerging technologies like virtual reality (VR), artificial intelligence (AI), and wearable sensors complement these interventions by offering automated feedback and facilitating more personalized rehabilitation plans. For instance, a digital therapeutic program that integrates exercise, nutrition, and robotic exoskeletons can be tailored based on age, body mass index, and recovery stage, improving functional outcomes and slowing weight loss in cancer patients. Moreover, remote CBT and group support provide psychological benefits, helping patients cope with stress, anxiety, and depression. Personalized education through telemedicine has proven effective, adapting platforms to meet patients’ needs based on factors such as age and education level.

Despite the promise of these technologies, challenges such as data privacy concerns, inconsistent digital literacy, and technical issues like poor internet connections must be addressed to fully realize the potential of remote cancer rehabilitation [34,35]. Nevertheless, telemedicine and robotic exoskeletons are essential components of modern cancer rehabilitation, offering patients the flexibility to engage in rehabilitation from home while improving functional recovery and overall quality of life [36]. Future research should focus on optimizing technology interfaces, standardizing implementation, and ensuring equitable access to these interventions for all patients.

### Technical foundations of robotic exoskeletons

#### Types and advancements in robotic exoskeletons for rehabilitation

Robotic exoskeletons are classified into active-assistive and passive types (Fig. 2) [37]. Active-assistive exoskeletons use sensors and actuators to provide powered support, making them ideal for patients with lower limb weakness or paralysis. These systems enable gait training and muscle recovery through precise force output (Table 2). In contrast, passive exoskeletons offer partial support and stability, making them suitable for early-stage rehabilitation or mild impairments [38].

**Table 2.** Comparison of exoskeleton technology parameters

Parameter	Powered exoskeleton	Passive exoskeleton
Drive mode	Motor-driven	Mechanical springs/elastic materials
Load capacity	High (can support full body weight)	Low (only assists local movements)
Control mode	Intelligent algorithms, sensor feedback	Purely mechanical control
Applicable cancer types	Breast cancer, colorectal cancer (lower limb rehabilitation)	Lung cancer (upper limb rehabilitation)

Both types of exoskeletons operate using dynamic control and kinematic modeling, combining mechanical frames with electric or pneumatic drives [39]. Real-time sensor feedback adjusts movement assistance for gait or daily activities [40]. The integration of AI and machine learning enables personalized motor support, adapting force and motion according to the patient’s needs (Table 2) [41]. Additionally, the development of flexible materials and wearable sensors has improved the portability and comfort of these devices. Electromyography-based systems monitor muscle activity to regulate assistance, preventing overcompensation and aiding motor recovery [42].

#### Application scenarios of robotic exoskeletons

Robotic exoskeletons play a key role in cancer rehabilitation, especially in gait recovery, cardiopulmonary training, and muscle endurance enhancement (Table 2). Gait recovery supports patients with neuromuscular deficits by restoring movement patterns via powered assistance. Cardiopulmonary training adjusts intensity/frequency to boost aerobic metabolism. Muscle endurance training combats atrophy from inactivity, while exoskeletons also provide stable early-stage support [43]. Compared to traditional therapy, exoskeletons reduce fatigue and build confidence. While direct studies in cancer are limited, exercise-based rehab improves strength and endurance and alleviates CRF and psychological distress in elderly patients [44]. Exercise also mitigates treatment-induced degeneration in cardiovascular, respiratory, and musculoskeletal systems, improving quality of life.

Exercise reduces systemic inflammation and improves redox balance, mitochondrial function, and immune regulation [45]. In breast cancer, it decreases tumor necrosis factor-α (TNF-α), interleukin-6 (IL-6), and CRP while enhancing metabolism and immunity [46]. A 10-week rehab at the National Cancer Center improved physical, emotional, and role functions and aerobic fitness [42]. Personalized RT using speed-load modeling enables precise intensity prescription, aiding recovery in breast cancer survivors. CORE provides exercise, nutrition, and psychological care. The integrated lifestyle modification for patients with cardiovascular toxicity or metabolic disorders enhance their quality of life more than a standalone program [47]. Although remote implementation requires more study, CORE shows strong clinical potential. Personalized aerobic and resistance programs also improve physical performance and HRQoL, as evidenced by walking and sit-to-stand tests [48,49]. In central nervous system (CNS) malignancies, neurorehabilitation maintains independence and safety. Compared to noncancer neurological rehab, CNS cancer rehab poses unique challenges [50,51]. In small cell lung cancer, tailored pulmonary rehab combining respiratory, gait, and aerobic training improves lung function, walking capacity, and quality of life [52].

### Mechanistic insights into exercise rehabilitation

CRP is typically managed through multimodal strategies combining psychological, behavioral, and pharmacological interventions. Among behavioral approaches, exercise stands out for its accessibility, low cost, and safety profile [53]. The 2019 International Multidisciplinary Roundtable recommended exercise for alleviating fatigue, depression, anxiety, and lymphedema in cancer patients [54]. However, exercise is not yet a primary treatment for CRP due to inconsistent and limited evidence. Broader meta-analyses suggest a moderate benefit of exercise. The 2019 National Comprehensive Cancer Network (NCCN) guidelines recommend physical activity for aromatase inhibitor-induced joint pain, highlighting its value for specific subgroups.

Exercise is a structured and planned physical activity, which involves aerobics, resistance and flexibility training. In oncology, it is emerging as a nonpharmacological pain management tool, particularly valuable amid the opioid crisis. Physiologically, exercise promotes endorphin release, improves joint mobility, reduces muscle tension, and enhances circulation. Tailoring programs to pain types (neuropathic, nociceptive, or neuroplastic) is essential in multimodal care [55]. Effective program design can follow the FITT principle (frequency, intensity, time, type). For example, strengthening and stretching exercises alleviate postoperative shoulder pain in HNC patients [56]. While exercise is well studied, nutrition is often overlooked. A review of pediatric cancer rehab showed functional gains from exercise, but lacked assessments of nutrition and feeding impairments. In remote rehabilitation, medical nutrition therapy remains underutilized. Future strategies should integrate exercise and nutrition to maximize outcomes [57].

In conclusion, exercise therapy has a strong physiological basis for cancer pain management but requires further research to optimize personalized interventions. Combining exercise with other modalities, especially nutrition, will enhance rehabilitation effectiveness.

#### Immune, inflammatory, and metabolic regulation

Moderate exercise reduces the risk of disease recurrence by enhancing immune function and reducing chronic inflammation. RT, in particular, lowers inflammatory markers such as CRP and IL-6 while increasing anti-inflammatory cytokines like IL-10 [58]. Robotic exoskeletons further amplify these effects by enabling high-intensity, feedback-driven training, offering precise immune regulation and targeted rehabilitation (Fig. 2).

Exercise also mitigates the metabolic dysfunction often induced by cancer treatments [59]. Bioinformatics analyses have revealed how exercise influences protein interactions and cellular signaling pathways. For instance, in elderly prostate cancer patients undergoing androgen deprivation therapy (ADT), RT has been shown to improve lean mass, skeletal muscle index, strength, and overall function. The benefits of exercise vary based on baseline function and age, underscoring the importance of personalized training regimens .

On a psychological level, exercise stimulates the release of neurotransmitters, improving mood, reducing anxiety and depression, and enhancing self-efficacy. This, in turn, promotes better treatment adherence and improves HRQoL. In patients with oral cancer, structured walking and resistance programs help alleviate fatigue and counteract the negative effects of chemotherapy and radiotherapy [60]. In prostate cancer, while both aerobic and RT improve HRQoL, the effects on fatigue and physical activity are more modest. Tailored exercise programs that consider the patient’s physical, emotional, and treatment status are recommended. Focus group discussions have highlighted the need for professional guidance and individualized plans to effectively manage symptoms and support long-term recovery [61]. Multimodal interventions have been shown to benefit diverse populations, including older and frailer patients, who experience substantial improvements in function, anxiety, and depression following intervention [62].

To conclude, it is evident that exercise therapy aids in modulating immune function, inflammation, metabolism and psychosocial factors that influence fatigue and emotional stress, as well as in improving quality of life and reducing recurrence risks.

#### Psychosocial and physical benefits of exercise in cancer rehabilitation

Exercise plays a crucial role in cancer rehabilitation by not only improving physical function but also enhancing psychological well-being, ultimately boosting HRQoL. For patients dealing with frailty and muscle wasting, interventions such as walking, RT, and nutritional support can improve physical function and daily living abilities. Personalized goal setting, based on behavior change models like gait speed and activities of daily living assessments, ensures tailored interventions. The FITT principle is used to progressively adapt exercise programs for optimal results [63].

Exercise has been shown to alleviate psychological symptoms, reduce fatigue, and enhance HRQoL. For example, aerobic training benefits lymphoma patients by improving health, reducing fatigue, and enhancing cardiovascular function and lean mass, regardless of treatment stage. Leukemia patients undergoing autologous hematopoietic stem cell transplantation (HSCT) also experience improved physical function and reduced fatigue with combined endurance–strength training. While short-term improvements in cardiopulmonary fitness, mobility, depression, and sleep are well documented, long-term effects on recurrence, pain, and social-emotional outcomes are still under investigation.

As cancer transitions into a chronic condition, maintaining physical activity post-treatment becomes essential. Many survivors experience a decline in activity levels that may not fully recover, but structured rehabilitation can foster lasting behavior change, improving long-term outcomes. Programs designed around behavioral theories equip survivors with skills to incorporate physical activity into daily life, helping them return to a sense of normalcy [64].

Exercise also fosters psychological resilience, enhances self-efficacy, and improves coping strategies, all of which contribute to reduced anxiety, depression, and enhanced treatment adherence. Professionally guided exercise programs, led by physical therapists and exercise physiologists, are not only safe but also effective in improving functional recovery and patient motivation [65]. Additionally, integrating exercise with biomarker assessments can further personalize cancer care [66].

In conclusion, exercise therapy offers psychosocial benefits for cancer patients, including reduced fatigue, improved mood, and enhanced HRQoL. By incorporating exercise into standard cancer care, a more holistic approach to recovery and long-term health maintenance is achieved.

### Behavior change theories supporting rehabilitation

#### Implementation of behavioral strategies

Behavior change techniques (BCTs) have proven effective in cancer rehabilitation, particularly for breast cancer survivors, by alleviating fatigue, depression, and anxiety while improving HRQoL. Among these techniques, BGA has shown success in sustaining long-term behavior change. BGA, based on operant conditioning, helps patients gradually increase activity within their tolerance, addressing daily challenges through structured engagement [67]. In cancer rehabilitation, incorporating BGA can lead to sustainable improvements in physical function and emotional well-being.

Cognitive behavioral cardiac rehabilitation (CBCR), which integrates CBT into cardiovascular rehab, provides a model for enhancing cancer rehabilitation strategies by reducing anxiety and improving long-term outcomes. However, the integration of behavioral strategies in cancer rehabilitation is still limited. Research indicates that personalized goal setting tailored to a patient’s prognosis and functional potential enhances engagement and adherence, leading to better outcomes [68]. Programs that incorporate a variety of BCTs, such as goal setting, task grading, and social support, have demonstrated higher effectiveness, although a solid theoretical foundation remains underdeveloped in many current interventions.

The use of robotic exoskeletons in cancer rehabilitation, combined with virtual support and wearable devices like Fitbits, enhances patient engagement by making exercise more enjoyable and sustainable. For older cancer survivors, who often face mobility limitations due to age, comorbidities, and treatment side effects, integrated interventions combining exercise, nutrition, and psychosocial support improve both mobility and quality of life [69]. Future research should focus on integrating these interventions, particularly for socially vulnerable groups, enhancing adherence to rehabilitation programs, and evaluating their economic feasibility to improve the quality and accessibility of cancer rehabilitation services.

## Clinical Application Prospects of Robotic Exoskeletons in Cancer Rehabilitation

### The role of robotic exoskeletons in enhancing cancer rehabilitation and functional recovery

**Fig. 3.**
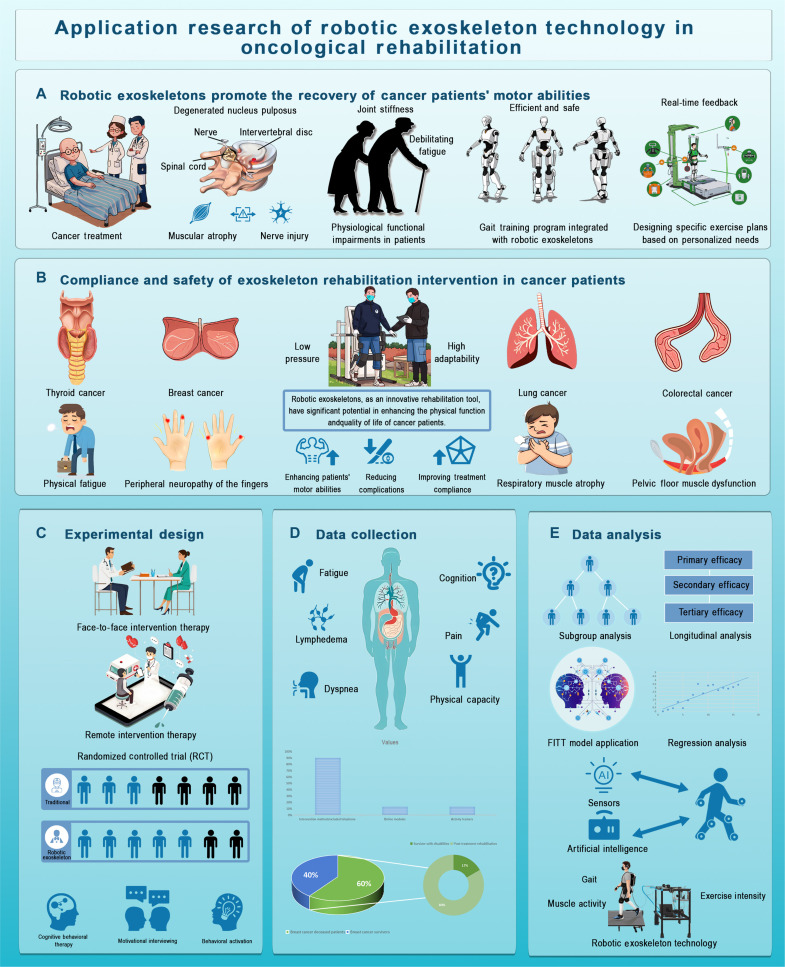
Robotic exoskeletons in oncological rehabilitation. (A) Promoting motor recovery. Robotic exoskeletons assist in restoring motor abilities by addressing issues like muscular atrophy, joint stiffness, and nerve damage. They support gait training, offer real-time feedback, and are tailored to individual patient needs for enhanced functional recovery. (B) Compliance and safety. This section discusses the adaptability of exoskeletons in treating various cancers, including thyroid, breast, lung, and colorectal. It highlights how these devices improve motor abilities, reduce complications, and boost patient compliance by addressing fatigue, muscle atrophy, and pelvic floor dysfunction. (C) Experimental design. The research utilizes a randomized controlled trial (RCT) to assess the effectiveness of robotic exoskeletons, incorporating both face-to-face and remote therapy sessions to ensure robust data validity. (D) Data collection. Key outcomes, such as fatigue, cognition, pain, and physical capacity, are captured using physiological assessments and patient-reported outcomes to comprehensively evaluate exoskeleton intervention effectiveness. (E) Data analysis. The data are analyzed through primary, secondary, and tertiary efficacy measures, with subgroup and longitudinal analyses. The use of AI and sensors to monitor gait, muscle activity, and exercise intensity is also emphasized to optimize rehabilitation outcomes using advanced technology.

Robotic exoskeletons are increasingly recognized for their role in enhancing functional recovery in cancer patients, particularly those experiencing muscle atrophy and neurological impairments due to treatment. These systems support gait, muscle strength, and joint flexibility, promoting recovery in both lower and upper limbs (Fig. 3) [70]. By dynamically adjusting walking patterns, exoskeletons help prevent fatigue and optimize training efficiency. For upper extremity dysfunction, they mimic natural arm movements, aiding in daily tasks and improving mobility. As patients progress, the level of assistance can be reduced, allowing for greater independence. Additionally, these exoskeletons offer psychological benefits by reducing anxiety and depression, boosting confidence, and improving overall quality of life [71]. Importantly, robotic exoskeletons should complement traditional physiotherapy rather than replace it. Integrated hybrid rehabilitation models, where exoskeletons provide standardized mechanical support and physical therapists tailor individualized training, are most effective in preventing both overtraining and undertraining. A multidisciplinary team, including rehabilitation physicians, physical therapists, and engineers, is essential to design safe and personalized intervention plans, supported by real-time feedback mechanisms to adjust intensity and monitor progress.

Adherence and safety are critical when using robotic exoskeletons in cancer rehabilitation, especially among elderly and frail patients. Physical activity improves daily function, reduces fatigue, enhances psychological well-being, and mitigates treatment complications [72]. For example, breast cancer patients with shoulder, upper limb, and hand movement disorders benefit from exoskeletons that restore flexibility and strength through precise joint control. Similarly, CIPN patients can undergo targeted gait training to restore balance, while lung cancer patients with respiratory muscle atrophy benefit from strengthening exercises post-surgery or radiotherapy. Colorectal cancer patients experience benefits from strengthening modules for the lower limbs and core, which also aid in bowel motility and relieve constipation.

Robotic exoskeletons offer the unique advantage of real-time, patient-specific modulation of support strength and movement patterns, enabling safer and more personalized interventions. For frail patients or those undergoing chemotherapy/radiotherapy, the systems provide low-impact, controlled environments that support gradual reconditioning. These systems demonstrate promising potential in improving cancer patients’ mobility, physical resilience, treatment adherence, and overall HRQoL [73].

### Evaluating robotic exoskeletons in cancer rehabilitation: RCT design and multidisciplinary approaches

This study used a randomized controlled trial (RCT) design to assess the effectiveness of robotic exoskeletons in cancer rehabilitation (Fig. 3). Participants were divided into an experimental group, which received rehabilitation supported by robotic exoskeletons, and a control group, which received traditional physical therapy or home exercise. The study covered various cancer types and stages, collecting multidimensional data on both physiological and psychological factors. Quantitative measures included cardiovascular endurance, gait analysis, and muscle strength, while psychological assessments and quality of life were evaluated using fatigue scores and HRQoL questionnaires. Qualitative data, such as patient interviews and satisfaction surveys, helped identify compliance issues and potential barriers to rehabilitation [74].

Robotic exoskeletons offer advantages over traditional rehabilitation methods, which often rely on manual therapist support, limiting their effectiveness and consistency. Exoskeletons, equipped with precise algorithms and sensors, dynamically monitor and adjust patient movements, improving training efficiency and stability. They also enable patients to train independently at home, extending rehabilitation access and increasing adherence [75]. However, high costs and limited availability remain obstacles to widespread adoption, requiring technological innovations and policy support.

The study also explored the effects of exercise interventions, either alone or combined with other strategies like behavioral guidance and education on reducing sedentary behavior. Many programs incorporated cognitive-behavioral techniques (CBT), behavioral activation, and motivational interviewing, which improved patient engagement through goal setting, social support, and reminders. For a more comprehensive approach, it is recommended that future studies combine robotic exoskeletons with traditional rehabilitation techniques such as strength training, aerobic exercise, and psychological support, using validated tools like the TUG test and the 5-time sit-to-stand test for functional recovery assessments [76].

Multicenter RCTs should also explore the impact of exercise interventions on biomarkers like CRP, IL-6, and TNF-α for breast cancer patients to validate broader applicability of results. Evidence supports that physical exercise improves fatigue, body image, and quality of life in cancer survivors, with remote interventions further enhancing engagement and activity levels. Integrating exercise with psychological counseling and optimizing intervention methods will be crucial for improving patient outcomes and engagement in cancer rehabilitation [77]. Combining RCT design with multidisciplinary approaches and standardized assessments will provide a comprehensive evaluation of robotic exoskeletons and traditional rehabilitation methods, offering more effective treatment options for cancer patients [78].

### Data collection and evaluation of robotic exoskeleton interventions in cancer rehabilitation

Across the studies reviewed, outcome evaluation tools varied, but most focused on physiological and cognitive aspects such as fatigue, pain, dyspnea, and physical capacity. Other studies assessed activity limitations and participation using disability-related indicators, including task performance, caregiver burden, and time-to-completion metrics. These assessments primarily relied on patient self-reports, providing insights into functional independence and overall life quality impact [79].

Preliminary findings indicate that robotic exoskeletons improve physical recovery and fatigue relief compared to traditional methods. Notable improvements were seen in gait stability, cardiopulmonary endurance, and lower limb strength. Multimodal rehabilitation plans, including robotic exoskeleton support, enhance therapeutic outcomes by reducing movement disorders and offering personalized rehabilitation [80]. Remote interventions, such as telephone or online platforms, have expanded accessibility for patients, particularly in underserved areas, and have been shown to improve HRQoL, especially by reducing fatigue and enhancing physical function [71,81]. For cancer patients, robotic exoskeletons provide a low-risk exercise environment that helps reduce CRF while improving mobility and confidence in rehabilitation (Fig. 3). Real-time feedback and adjustable parameters further personalize rehabilitation, although high costs and limited availability are ongoing challenges [30]. Robotic exoskeletons foster physical function improvement and better HRQoL, contributing to functional recovery and treatment adherence, while supporting fatigued patients in increasing activity levels without additional strain, significantly alleviating CRF [82].

For a more comprehensive evaluation, both quantitative and qualitative indicators should be combined. Standardized motion parameters and safety monitoring are essential for assessing primary outcomes such as the 6-min walk test (6MWT), fatigue scores, and adherence rates. Additional assessments like grip strength, the Berg balance scale, and endurance tests offer deeper insights into physical recovery. Psychological assessments (e.g., anxiety and depression scales), cardiopulmonary tests, and muscle strength evaluations are crucial for understanding the multidimensional effects of robotic exoskeleton interventions [83]. Including information about inflammatory markers, insulin, fasting glucose, and lipid levels, together with information on physical activity and psychological parameters, will yield a more comprehensive evaluation of the intervention effect.

### Methods of data analysis

Subgroup analyses can identify differential responses to exoskeleton interventions, such as comparing frail versus nonfrail patients. Longitudinal studies evaluate sustained rehabilitation effects across cancer types and age groups [62]. The FITT model assesses exercise intervention efficacy on functional recovery and quality of life [84].

Multiple regression models can analyze the impact of physical exercise on quality of life and fatigue, while considering factors such as gender, age, and disease stage, revealing the relationship between patient characteristics and rehabilitation outcomes, and achieving targeted rehabilitation. In addition, regression models can evaluate the correlation between changes in biomarkers and rehabilitation outcomes, especially the impact of exercise intervention on biomarkers. To standardize the assessment of rehabilitation outcomes, an international Delphi consensus study is developing a core outcome set (COS) for lung cancer rehabilitation. This study will use systematic reviews, focus groups, and multiple rounds of surveys to identify and prioritize key outcome indicators, which will provide a unified framework for clinical practice [85]. It emphasizes the need to consider participant characteristics and exercise types when designing interventions. Recruitment barriers such as long-distance travel, insufficient information, and underestimation of treatment side effects can affect research results, and recruitment strategies need to focus on these factors to increase participation rates [86]. Integrated analysis of single-cell RNA sequencing can be used to deeply understand the individual response of cancer patients to rehabilitation intervention, providing a basis for precision rehabilitation. Sensors and AI are integrated into the exoskeleton of robots to achieve data-driven rehabilitation optimization, real-time monitoring of gait, exercise intensity, and muscle activity, helping therapists gain a deeper understanding of progress and adjust personalized plans. Smart materials can optimize the interaction between the exoskeleton and biological tissue, reducing inflammatory response . Lastly, remote medical intervention combined with activity trackers and real-time feedback provides personalized rehabilitation methods, continuously monitors relevant indicators to dynamically adjust plans, improves rehabilitation outcomes, and supports future research.

Through the application of these data analysis methods, this research aims to explore the impact of robotic exoskeleton interventions on cancer patients’ rehabilitation outcomes, laying the foundation for personalized treatments and further optimizing rehabilitation through data-driven approaches [87].

## Clinical Application Prospects of Robotic Exoskeletons in Cancer Rehabilitation

### Enhanced physical recovery and fatigue relief with exoskeleton intervention

Exercise, including aerobic and RT, has been shown to improve bone mineral density (BMD) and muscle strength in cancer survivors. Weight-bearing exercises stimulate osteoblast activity, increasing BMD, while calcium and vitamin D supplementation further supports bone recovery. These interventions also improve HRQoL and reduce bone-related events [88]. Integrating robotic exoskeleton systems into rehabilitation programs enhances these benefits by allowing real-time adjustments based on patients’ physiological responses and fatigue levels, improving safety and individualization [89]. RT, combined with robotic exoskeletons, helps alleviate CRF, enhances muscle strength, and reduces inflammation, improving physical function [90]. This combination optimizes training intensity, reduces injury risk, and is particularly beneficial for cancer survivors with severe fatigue or poor physical condition.

### Increased comfort and confidence with exoskeletons

**Fig. 4.**
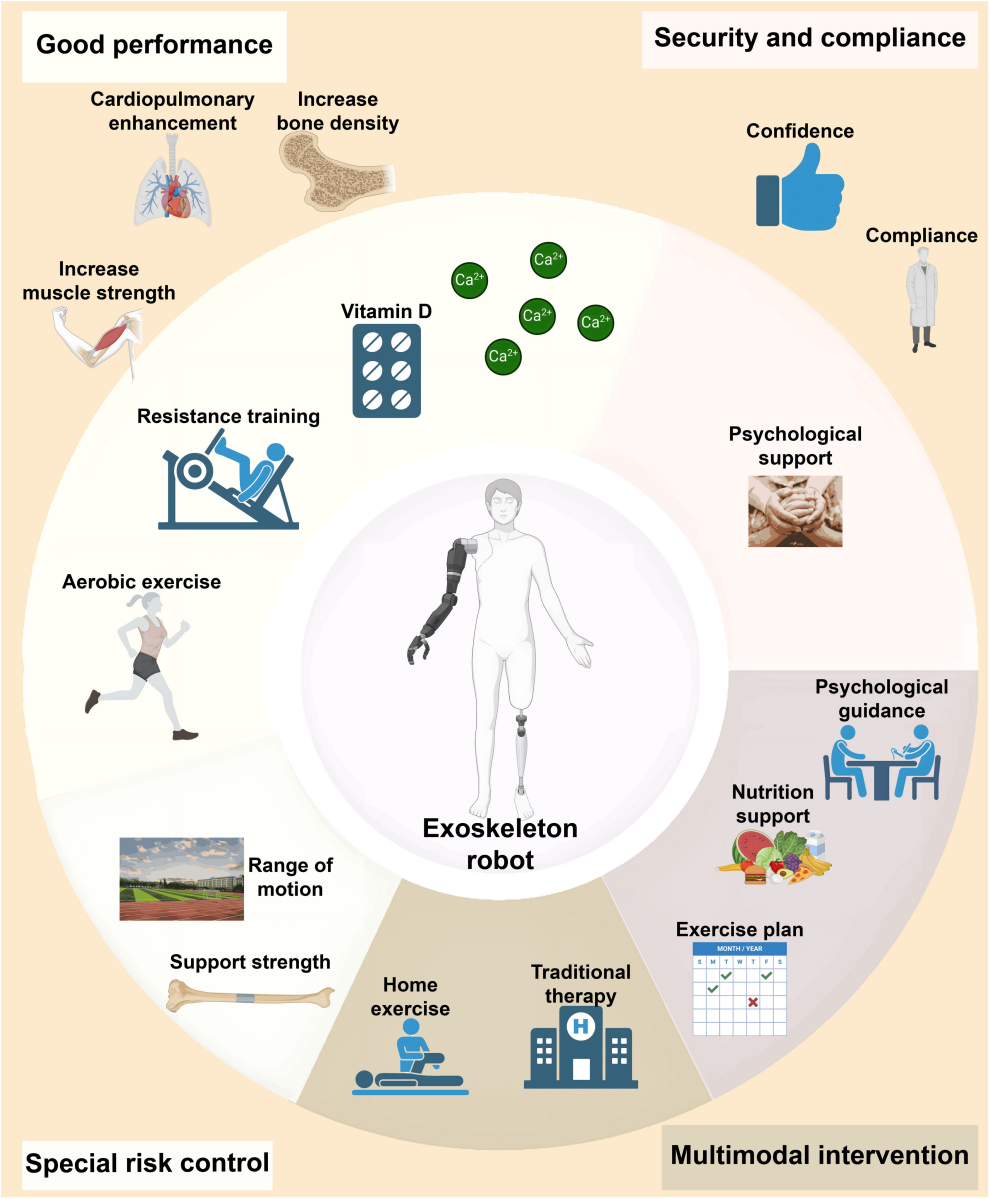
This figure presents a framework for multimodal interventions to enhance the performance, security, and compliance of patients using an exoskeleton robot. It highlights aspects such as improving physical performance (cardiopulmonary, muscle strength, bone density, RT, and aerobic exercises), the role of vitamin D and calcium, patient adherence and confidence during rehabilitation, psychological support, and special interventions (range of motion, strengthening bones, home exercises, and traditional therapy). The exoskeleton robot integrates these elements to optimize patient outcomes.

Multidisciplinary rehabilitation programs improve functional independence, reduce fatigue, and enhance HRQoL in cancer patients, supporting broader use of robotic exoskeletons. These programs enhance gait speed and muscle strength and reduce fall risk, especially in elderly patients [91]. Regular exercise, including aerobic activity, RT, and yoga, improves physical and emotional health. Nurse-led interventions addressing CRF, pain, cognitive deficits, and mental health further boost function and quality of life. Cancer-related disabilities often reduce daily activity, life satisfaction, and work participation, leading to increased psychological stress and social isolation. Psychological support is crucial in exercise rehabilitation. Robotic exoskeletons improve patient confidence, mobility, and adherence by providing real-time feedback on progress, helping to overcome exercise-related fear and enhancing HRQoL [92,93]. Psychological support is essential in exercise rehabilitation. Robotic exoskeletons enhance patient confidence by improving mobility and providing real-time progress feedback (Fig. 4), helping overcome exercise-related fear and boosting adherence.

### Safety and adherence

#### Special risk control for bone metastasis patients

Safety is a top priority in rehabilitation, and robotic exoskeletons play a crucial role in minimizing risks during training. Their design optimizes motion range and dynamically adjusts support, reducing the likelihood of fractures or muscle strains, particularly in bone metastasis patients [94]. While mild fatigue or discomfort may occur initially, these effects typically resolve with adaptation. Ongoing advancements in technology and user experience further enhance the safety of exoskeletons, solidifying their importance in cancer rehabilitation.

For bone metastasis patients, whose bones are fragile and prone to fractures, traditional rehabilitation can be challenging. Robotic exoskeletons offer precise, customizable support, adjusting training modes and intensity to protect bones while improving muscle strength and mobility (Fig. 4) [95]. Low-intensity exoskeleton-assisted exercises can help reduce pain, promote bone metabolism, delay disease progression, and enhance quality of life. Additionally, injectable multifunctional composite hydrogels, which promote bone regeneration through immune regulation and osteogenesis, provide promising adjuncts to protect bone health during exoskeleton training [96].

#### Exoskeleton adherence analysis

In the rehabilitation of advanced cancer patients, the introduction of exercise interventions must take into account the complex clinical manifestations and the safety concerns caused by multiple symptoms [6]. Personalized guidance is crucial for improving compliance, especially for elderly patients who face treatment compliance challenges due to physical decline and increased complications. Optimizing exoskeleton design to reduce physical burden and enhance personalized guidance is an effective strategy for improving compliance. Elderly patients often suffer from complications and limited mobility. It is necessary to adjust the intensity and frequency of exercise according to their health status, especially when dealing with CRF and bone health damage [97]. Supervised exercise plans should take into account the patient’s condition and avoid the risks of high-intensity exercise. Personalized guidance can ensure the safety and effectiveness of rehabilitation interventions. Studies have found that robotic exoskeletons enhance both safety and adherence in cancer patients’ rehabilitation. For patients with bone metastases, exoskeleton system design prevents fracture risk by precisely adjusting support and range of motion. Furthermore, its user-friendly design and ease of operation improve patient engagement, and the intelligent feedback system adjusts training parameters in real time based on progress, enhancing patient confidence and persistence, prolonging intervention time, and improving rehabilitation outcomes [98]. Remote medical intervention also improves participation and compliance, with real-time interaction and personalized feedback to increase patient control, especially for remote or mobility impaired patients. Remote medical provides convenience, but is still limited by technological and psychological factors. Future improvements should focus on the ease of exoskeleton system operation and user education to further enhance intervention effectiveness.

### Synergistic effects of multimodal interventions in cancer rehabilitation

Combining nutritional support, psychological counseling, and exercise enhances rehabilitation, particularly in elderly patients’ physical recovery and independence (Fig. 4) . Integrating robotic exoskeletons with traditional exercise further improves mobility, adherence, and patient participation, offering comprehensive support [99]. Programs like Sheffield’s “Active Together” address physical, nutritional, and psychological needs, leading to improved clinical outcomes and quality of life [100]. Similarly, combined exercise programs in HNC enhance physical activity and HRQoL, with efforts to optimize participation [101]. Multidimensional rehabilitation (MDR) in breast cancer patients outperforms home-based exercises, boosting endurance and activity after supervised training. Community rehabilitation involving physical and occupational therapy improves physical and mental health, function, and social participation across cancers, supporting its inclusion in standard care [102]. The “iCan” program also shows that patient-centered MDR improves fitness and reduces fatigue.

Future cancer rehabilitation will rely on multimodal approaches that combine exercise, nutrition, psychological support, and pharmacology for maximal effect. Robotic exoskeletons complement these interventions by enhancing movement stability, reducing anxiety, and improving metabolic efficiency when combined with nutritional support. For example, RT with calcium and vitamin D supplementation improves bone density and mobility, while psychological support alleviates fracture fear, boosting adherence and providing multidimensional therapeutic benefits. When compared to traditional methods, robotic exoskeletons enhance rehabilitation, leading to long-term improvements in functional abilities and quality of life. Multimodal rehabilitation programs are more effective in reducing chemotherapy-induced complications, such as nausea and fatigue, and are associated with reduced hospitalization rates, particularly in elderly cancer patients. The combination of psychophysical interventions and modern technologies like exoskeletons promotes functional recovery and improves quality of life. Compared to single interventions, combining home exercise programs with traditional treatments is more efficient in improving physical function and psychological well-being in thyroid cancer patients. However, challenges such as high costs and limited access to technical equipment remain. Future efforts should focus on developing cost-effective interventions to expand their reach and applicability to a larger patient population [103].

## Translational Challenges and Innovation Pathways

### Economic challenges and future directions for robotic exoskeletons in cancer rehabilitation

Remote medical interventions, including robotic exoskeletons, offer advantages in cancer rehabilitation by improving accessibility, but their high cost remains a major challenge. While telephone and internet-based interventions are more affordable, the use of advanced systems like exoskeletons and activity trackers increases financial burdens on both patients and healthcare institutions [104]. Policy support, medical insurance coverage, and resource-sharing models, such as community rehabilitation centers with shared equipment, are considered effective solutions to reduce costs and ensure sustainable implementation.

**Fig. 5.**
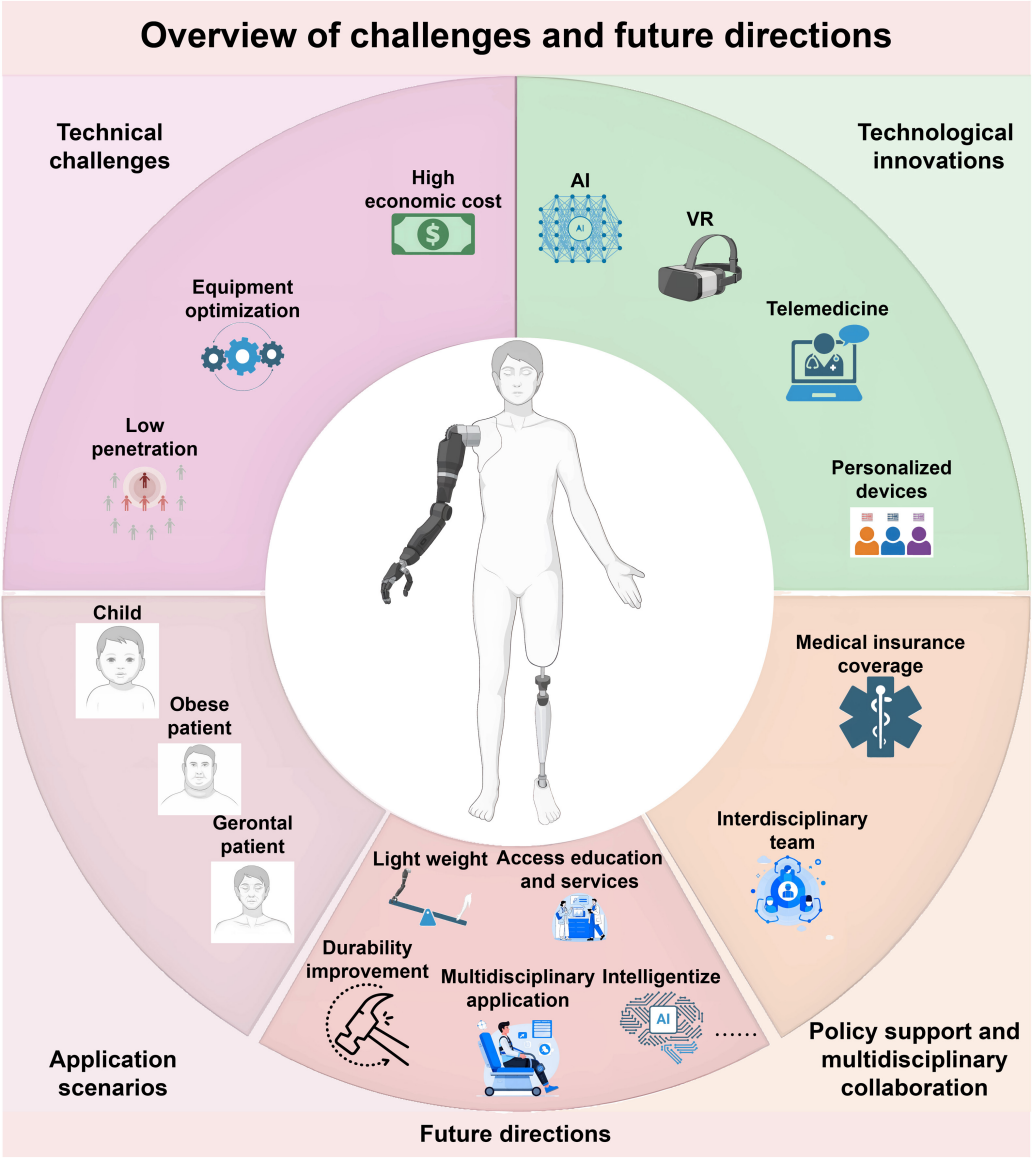
This figure outlines the challenges and future directions for exoskeleton robots. It covers key obstacles such as high costs, the need for equipment optimization, and limited population penetration, especially for groups like children, obese patients, and the elderly. Technological advancements like AI, VR, telemedicine, and personalized systems are highlighted as solutions to these challenges. The figure also explores application potential in various patient groups and emphasizes the importance of improving exoskeleton durability, reducing weight, and increasing access to healthcare services. Future directions include enhancing multidisciplinary applications, intelligent integration, and ensuring medical insurance and policy support.

The high cost and uneven distribution of rehabilitation resources hinder the widespread adoption of robotic exoskeletons, particularly for elderly cancer patients who face economic barriers and limited access to medical resources [105]. There is a need for cost-effective exoskeleton systems that meet diverse demands and lower technological barriers (Fig. 5) [106]. For obese patients, who often struggle with traditional exercise due to joint stress and muscle weakness, robotic exoskeletons offer added support, reducing pain and enhancing mobility. However, issues such as exoskeleton weight and usability may limit their adaptability for obese patients, requiring further design optimization. Emerging technologies like microfluidic vascular chips for thrombosis prevention and high-resolution electron microscopy for analyzing exoskeleton materials offer promising opportunities to improve biocompatibility and performance [107].

### Personalization and precision medicine

#### Data-driven personalized rehabilitation programs

Future robotic exoskeletons must be personalized and intelligently adapted to individual patient needs, physical conditions, and rehabilitation progress. This requires advanced robot control systems, sensor technologies, and AI algorithms to provide precise motion assistance tailored to each cancer patient’s unique treatment history and rehabilitation requirements (Fig. 5) [108]. Integrating precision medicine and genomics allows customization of exoskeleton movement patterns and training intensities based on individual differences. For example, patients with decreased bone density after radiation therapy may require specialized bone weight-bearing training support, while patients with nerve damage caused by long-term chemotherapy can optimize the rehabilitation process by real-time monitoring of nerve responses and adjusting assistive forces through specific sensors. This personalized robotic exoskeleton system not only enhances the precision of treatment but also reduces potential risks associated with inappropriate treatment methods. As intelligent algorithms and data analysis technologies continue to evolve, future exoskeleton systems will be able to further optimize their adaptability and customization levels through big data and patient feedback [109].

Based on precision medicine and data analysis, personalized rehabilitation plans have become an emerging trend in cancer rehabilitation. Robot exoskeletons use sensors to collect real-time data, combined with AI algorithms to dynamically analyze patients’ physiological states, and customize rehabilitation paths for patients with different conditions and rehabilitation goals. For example, for bone metastasis patients, the exoskeleton system’s support strength can be adjusted to reduce the risk of fractures; for post-surgical patients, the exoskeleton can safely increase gait training intensity to achieve muscle rebuilding and functional recovery.

#### Precision medicine for robotic exoskeletons

Future cancer rehabilitation should adopt a data-driven, personalized model that tailors interventions to patients’ specific conditions and goals. Precision medicine integrating disease characteristics and functional status will optimize recovery by dynamically adjusting rehabilitation strategies [110]. Research should develop plans based on cancer type, stage, exercise tolerance, and individual health profiles, enabling personalized exercise prescriptions for improved outcomes. Combining physical exercise, psychological support, and nutrition needs a multidisciplinary approach to enhance rehabilitation. Integration of AI, telemedicine, and VR can further boost efficiency and patient adherence. Incorporating multiple biomarkers will refine exercise interventions and support long-term recovery [111]. Obesity poses unique rehabilitation challenges due to chronic inflammation limiting high-intensity exercise. Systems designed with higher load-bearing capacity and knee stability, equipped with smart sensors for dynamic support adjustments, enable low-impact exercise and reduce tissue stress for long-term benefits [112]. For patients undergoing cellular therapies, such as chimeric antigen receptor (CAR) T cell therapy, exoskeletons can enhance gait stability, lower limb strength, and reduce CIPN [113]. Tests such as 6MWT and HGS help ensure safe recovery. Exoskeletons provide low-risk, noninvasive options for patients weakened by treatment toxicities [71,114]. Childhood cancer survivors face challenges due to long-term treatment effects, including CIPN and muscle weakness. Robotic exoskeletons provide dynamic support for physical therapy interventions, improving gait and muscle strength. Integrating real-time data monitoring optimizes treatment plans, enhancing patient compliance and effectiveness [115]. Beyond short-term recovery, robotic exoskeletons offer long-term support for childhood cancer patients, helping maintain functional capacity and adjusting training parameters as the patient progresses. These exoskeleton systems provide visual feedback and are integrated with gamified training programs, boosting rehabilitation confidence and patient motivation [116].

### Multidisciplinary collaborative rehabilitation services

#### Data-driven personalized bone health management and synergistic effects of robotic exoskeletons

Future cancer rehabilitation research should focus on developing data-driven, personalized bone health management plans by integrating real-time data monitoring with AI. This will allow exercise programs to be dynamically adjusted to meet individual patient needs. Rehabilitation programs will become more effective and enable better implementation when multidisciplinary teams, e.g., physical therapists, nutritionists and oncologists collaborate. At the policy level, increased support for patient education is crucial to improving engagement and adherence, helping patients understand the importance of bone health management. Additionally, innovations in rehabilitation programs can explore the optimal integration of robotic exoskeletons with RT. By combining dynamic monitoring with data analysis, training programs can be better optimized. AI-driven exoskeletons can adjust training parameters in real time, providing more precise rehabilitation plans, and offering important potential for functional recovery in cancer survivors.

#### Core role of multidisciplinary collaboration in rehabilitation

Future rehabilitation models should prioritize multidisciplinary collaboration, integrating the expertise of physical therapists, occupational therapists, mental health professionals, and data scientists. Robotic exoskeletons can serve as a central component, working alongside telemedicine, VR, and other assistive technologies to provide comprehensive support for patients. To expand access, reducing the cost of exoskeleton systems and optimizing their design will enable more healthcare institutions to offer this technology, benefiting a wider range of cancer patients, particularly pediatric ones [117]. Interdisciplinary teams are crucial, especially when patient prognosis is uncertain. Optimizing collaborative mechanisms can improve intervention effectiveness. For example, integrating clinical medicine with engineering technology can help incorporate exoskeleton systems into the rehabilitation process, validating their applicability across different cancer patient populations. Multidisciplinary collaboration is essential for cancer rehabilitation, driving technological innovation and improving patient outcomes. By defining clear roles within teams and ensuring ongoing communication, the integration of diverse expertise can create personalized rehabilitation solutions. With support from policy, technological collaboration, and educational outreach, this model can expand to more patient groups, promoting functional and quality-of-life recovery [118].

#### Comprehensive rehabilitation interventions for elderly cancer patients

In the rehabilitation of elderly cancer patients, the integration of social support, psychological intervention, and medical care by multidisciplinary teams is crucial. This holistic approach enhances treatment outcomes, with the synergistic effects of social and psychological care being particularly important. Exercise rehabilitation should be optimized to combine physical training, psychological counseling, and nutritional support, offering comprehensive recovery and improving physical function, mental health, and quality of life. Similarly, with the growing use of cell therapies like hematopoietic stem cell transplantation and CAR T cell therapy for hematologic malignancies, patient survival rates have improved, yet these therapies often result in specific toxic reactions such as cytokine release syndrome and neurotoxic syndrome, leading to severe functional impairment. Patients typically experience fatigue, muscle weakness, and restricted mobility [119]. A multidisciplinary approach is essential in addressing these rehabilitation challenges. In the context of skeletal health, optimizing bone density and managing fractures in cancer patients requires policy support and patient education. Ensuring health insurance covers bone density testing and bone-protecting medications, alongside educating patients on exercise regimens and nutritional guidelines, can improve rehabilitation outcomes and patient participation in recovery programs.

Finally, the integration of robotic exoskeletons in rehabilitation relies on multidisciplinary collaboration. Clinicians tailor personalized rehabilitation plans, engineers ensure system compatibility, and physical therapists adjust training regimens based on patient progress. Psychologists play a key role in addressing the emotional challenges of using exoskeletons, which enhances adherence and overall effectiveness. Future research may explore combining exoskeletons with precision drug delivery systems to create more synergistic rehabilitation approaches, improving metabolic regulation and treatment outcomes for cancer patients [120].

#### Enhancing patient education, service accessibility, and multidisciplinary collaboration in cancer rehabilitation

Patient acceptance of cancer rehabilitation services largely depends on the quality and availability of information. Currently, many patients only learn about these services by chance due to inconsistent understanding among medical teams regarding the definition, goals, and value of rehabilitation, which limits service promotion and uptake. Additionally, fragmented healthcare models and the lack of standardized rehabilitation practices hinder effective information dissemination. To improve participation, healthcare teams must enhance patient education by clearly outlining rehabilitation goals, program structures, exercise guidelines, nutrition, and prevention strategies. Strengthening bone health through policies like expanded bone density screening and insurance coverage can also support rehabilitation access. Multidisciplinary collaboration is essential for developing patient education programs that maximize service effectiveness.

Optimizing referral and triage systems is equally important to address the complex needs of cancer patients. Most centers use functional assessments for specialized treatments, and dynamic adjustments to rehabilitation plans can improve patient safety and outcomes. However, limited oncology clinic awareness restricts referral rates. Tools like the Cancer Rehabilitation and Exercise Screening Tool (CREST) help identify patients who are sedentary or functionally impaired, improving resource allocation. Personalized exercise rehabilitation triage systems, such as the PERCS study, offer tailored rehabilitation pathways and proved adaptable during the COVID-19 pandemic, improving both physical and mental health outcomes [121].

The successful implementation of robotic exoskeletons in cancer rehabilitation also requires policy support and interdisciplinary collaboration [122,123]. Government initiatives such as subsidies and streamlined market access can accelerate adoption, while collaboration between healthcare providers, engineers, and data scientists optimizes system performance. Patient education programs further enhance understanding and acceptance of these technologies, especially in pediatric cancer rehabilitation, where improved mobility can enhance quality of life.

Multidisciplinary collaboration is also key in telemedicine interventions. Research shows that the joint participation of nurses, physical therapists, psychologists, and social workers improves rehabilitation outcomes. Telemedicine connects patients with professional teams through virtual platforms, offering psychological support, pain management, nutritional advice, and functional training. By combining psychological support with physical function training, patients can better cope with both physical and psychological challenges, achieving overall improvements in health.

### Integration of new technologies: Remote healthcare, AI, and VR for efficient rehabilitation

Integrating robotic exoskeletons with remote healthcare, AI, and VR offers personalized and efficient rehabilitation for cancer patients. Robotic exoskeletons enhance physical strength and motor functions, while VR supports physical and cognitive training, improving engagement and outcomes. AI dynamically adjusts intensity and training modes using real-time data, optimizing precision and adherence. Wearable exoskeletons combined with biosensors enable continuous monitoring, and emerging technologies like light-sensitive nanomaterials allow real-time tumor tracking and adaptive rehabilitation [124,125]. Remote healthcare enhances access, especially for patients with mobility limitations, by enabling real-time interaction with professionals and ensuring continuous treatment [81]. These systems guide safe, controlled exercises, and VR further boosts adherence and motivation. Future rehabilitation may combine robotic exoskeletons with motion analysis, nutritional strategies, and AI-driven personalization via cloud computing to optimize treatment and support both physical and psychological recovery [126,127]. Pediatric cancer survivors especially benefit from remote care tailored to patient and family needs. Research should prioritize evaluating cost-effectiveness and identifying optimal multimodal intervention. In summary, integrating robotic exoskeletons with AI, VR, and remote healthcare improves mobility, treatment adherence, and quality of life for cancer patients. Future efforts should aim to reduce costs, enhance user experience, and expand adoption, particularly for pediatric and underserved populations, to promote functional recovery in chronic conditions [20,128].

## Conclusion

Research on robotic exoskeletons in cancer rehabilitation mainly focuses on short-term effects, but future studies should prioritize long-term outcomes like quality of life, muscle strength, and system safety for sustainable clinical use. Incorporating features such as biosignal monitoring, drug delivery, and psychological assessments will create a more comprehensive rehabilitation system, improving cancer patient outcomes. With advancements in AI, exoskeletons can adapt to individual abilities, optimizing rehabilitation. However, challenges like high costs and complexity remain, and future research should aim to make exoskeletons lighter, smarter, and more durable, reducing the economic burden on patients and healthcare providers. Integrating remote healthcare, AI, and VR into a multimodal approach will improve rehabilitation efficiency, especially in resource-limited settings or for patients with mobility restrictions, enabling personalized guidance at home. Personalized rehabilitation plans based on real-time data will optimize treatment and outcomes. Policy support and collaboration are essential for widespread clinical adoption of this technology.

As manufacturing technologies improve, the cost of exoskeletons will decrease, making them more accessible to both healthcare centers and homes. Increased R&D investment will expand availability to community hospitals and home settings. Despite their potential, cost and policy constraints hinder widespread adoption. Future research should focus on assessing their effectiveness in various cancer populations, especially those with bone metastasis or long-term survival. Integration of big data and remote monitoring will optimize interventions and reduce costs. Resource-sharing models, like exoskeleton system sharing in community centers, could improve access and lower costs.

The success of robotic exoskeletons in cancer rehabilitation depends on technological advancements and policy support. Systemic change will maximize their potential, helping combat functional decline and improving quality of life. Future research should validate long-term clinical effects and integrate exoskeletons into multimodal rehabilitation programs. Combining exoskeletons with physical therapy, nutritional support, and psychological care will improve functional independence, especially in elderly patients. Personalizing exoskeleton systems will address individual needs and enhance long-term adherence. Large-scale, multicenter studies are needed to verify long-term outcomes and standardize measurement tools for better comparability. Optimizing system design and reducing costs will increase accessibility. In summary, robotic exoskeletons have great potential for personalized cancer rehabilitation and, with continued technological progress and interdisciplinary collaboration, could become integral to cancer care.
